# Supplementary material for: Population dynamics of generalist and specialist strategies under feast-famine cycles
Source: PLoS Comput Biol. 2026 Apr 30;22(4):e1014265. doi: 10.1371/journal.pcbi.1014265 (PMC13155689; doi:10.1371/journal.pcbi.1014265)
Supplement: S1 Text — (PDF) [file pcbi.1014265.s008.pdf]

Supplementary Information for *Population dynamics of generalist and specialist strategies under feast-famine cycles*

Rintaro Niimi[1], Chikara Furusawa[2,3*], Yusuke Himeoka[2*]

1 Department of Biological Sciences, Graduate School of Science, The University of Tokyo, Bunkyo-ku, Tokyo, Japan
2 Universal Biology Institute, Graduate School of Science, The University of Tokyo, Bunkyo-ku, Tokyo, Japan
3 Center for Biosystems Dynamics Research, RIKEN, Chuo-ku, Kobe, Japan

* furusawa@ubi.s.u-tokyo.ac.jp (CF), yhimeoka@ubi.s.u-tokyo.ac.jp (YH)

Contents

1 Robustness to the specific functional form of the convex resource-use trade-off 2

2 Evolutionary invasion analysis. 5

3 Evolutionary invasion analysis in $N = 2$ case 6

4 Analytical solution for the temporally averaged population under generalist-dominant condition 9

5 Consideration of Multiple Nutrients 14

6 Consideration of Concentration-dependent Growth Rates 16

7 Dependence of results on the growth-death trade-off function 19

8 Simulation with asymmetrically sampled environments 22

9 Population dynamics with an asymmetrically specialized generalist 23

10 Robustness of the generalist-specialist transition against changes in the trade-off shape 24

11 Simulations allowing access to sub-optimal interior phenotypes 25

1 Robustness to the specific functional form of the convex resource-use trade-off

In the main body of the paper, we imposed the following constraint on the resource-use trade-off:

$$\left(\prod_{e=1}^{E} \mu_{i,e}\right)^{\frac{1}{E}} = \bar{\mu}, \tag{1}$$

where $\mu_{i,e}$ denotes the growth rate of phenotype i under environment e, and $\bar{\mu}$ is the prescribed geometric mean growth rate across all E resources.

To test the robustness of our findings, we further examined a canonical nonlinear convex trade-off function originally introduced in a previous study [1]:

$$\sum_{e=1}^{E} \mu_{i,e}^{\alpha} = m, \tag{2}$$

where α and m are the positive constants. Parameter α modulates the curvature of the trade-off function, governing whether it is convex ($\alpha < 1$) or concave ($\alpha > 1$), while $\alpha = 1$ yields a linear trade-off.

We adopted $\alpha = 0.8$ in the following simulations. As described in the main text, we performed simulations with three phenotypes (phenotype 1,2, and 3) and two environmental conditions (environment A and B). The growth rates of the three phenotypes were set as follows:

$$\mu_{1,A} = \mu_{3,B} = (0.9m)^{\frac{1}{\alpha}}, \mu_{2,A} = \mu_{2,B} = (0.5m)^{\frac{1}{\alpha}}, \mu_{3,A} = \mu_{1,B} = (0.1m)^{\frac{1}{\alpha}}. \tag{3}$$

Phenotype 2 has a generalist-like strategy, while phenotype 1 and 3 are specialists adapted to Environment A and B, respectively.

Using this setup, we ran simulations and obtained results that were consistent with the results of the resource-use trade-off specified by Eq (1) (see main text). Namely,

- The dominant phenotype was determined by the growth-death ratio (Fig A).
- Increasing the mean and variance of $\Delta\tau$ increases the time average of the population of specialists (Fig A and Fig B).

We also examined a weak (concave) trade-off case using Eq (2) with $\alpha = 1.2$. The results are shown in Fig C: no generalist-specialist transition occurs, and the generalist remains dominant.

Under weak resource-use trade-offs, prior frameworks predict that generalists remain superior, because the trade-off constraint makes a balanced allocation of growth rates across environments optimal [1]. Importantly, in our model, adding the growth-death trade-off does not induce a generalist-specialist switch. When we increase a phenotype's growth rate in an environment, its death rate also increases under the trade-off. Thus, any growth advantage of a specialist is often accompanied by a larger loss during famine. Overall, this tends to favor generalists rather than specialists.

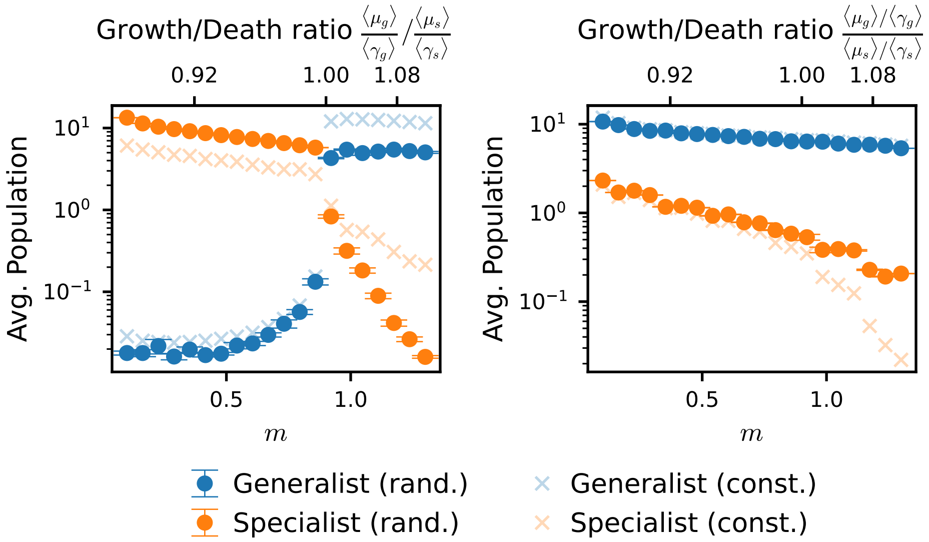

Fig A. Temporal average of the population with constant $\Delta\tau$. The average population numbers are shown as a function of the parameter m. The model with one generalist and two specialists is simulated for (a), while there is only a single specialist adapted to environment A for (b). Crosses ($\times$) denote results with constant nutrient supply intervals ($\Delta\tau_{\mathrm{const.}} = 100$), whereas circles ($\bullet$) denote those with gamma-distributed intervals ($\Delta\tau \sim \Gamma(2, 50)$). We simulated up to $t = 3.0 \times 10^5$ and averaged the populations after $t = 2.5 \times 10^5$. For each m, we ran 10 simulations and calculated the average population. The error bar indicates the standard error. All other parameters were identical to those used in Fig 2 in the main text.

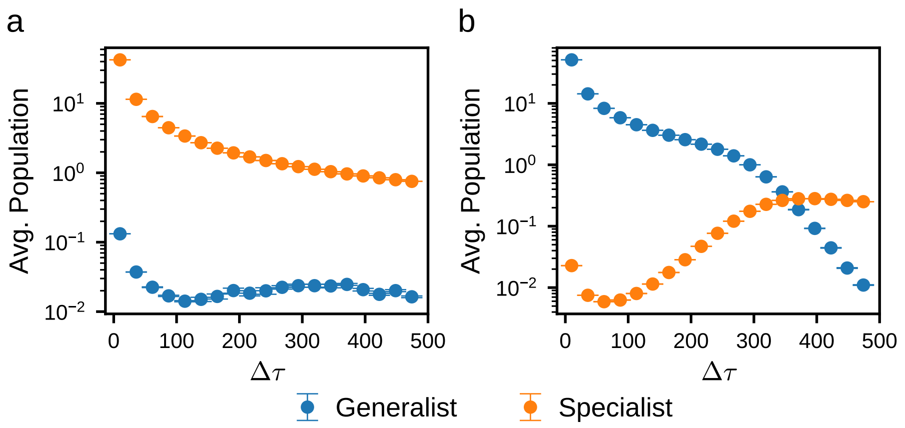

Fig B. Temporal average of the population as a function of $\Delta\tau$. Changes in the average populations as the parameter $\Delta\tau$ is varied in simulation with one generalist and two specialists. The case with $m = 0.5$ is shown in (a) and the case with $m = 1.5$ is shown in (b). We simulated up to $t = 3000 \times \Delta\tau$ and averaged the populations after $t = 2500 \times \Delta\tau$. $\Delta\tau$ is constant value. For each $\Delta\tau$, we ran 10 simulations and calculated the average population. The error bars indicate the standard error. All other parameters were identical to those used in Fig 4 in the main text.

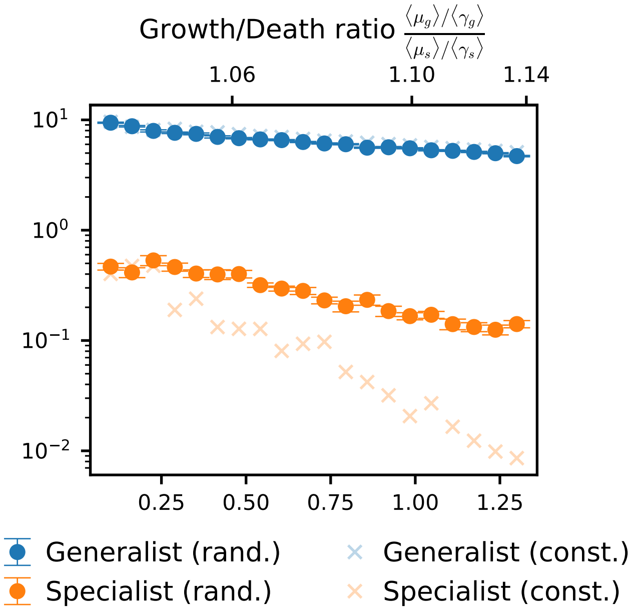

Fig C. Temporal average of the population with weak resource-use trade-off. The average populations are shown as a function of the parameter m, with $\alpha = 1.2$. The model with one generalist and two specialists is simulated. Crosses ($\times$) denote results with constant nutrient supply intervals ($\Delta\tau_{\text{const.}} = 100$), whereas circles ($\bullet$) denote those with gamma-distributed intervals ($\Delta\tau \sim \Gamma(2, 50)$). We simulated up to $t = 3.0 \times 10^5$ and averaged the populations after $t = 2.5 \times 10^5$. For each m, we ran 10 simulations and calculated the average population. The error bars indicate the standard error. All other parameters were identical to those used in Fig 3 in the main text.

2 Evolutionary invasion analysis.

To clarify why the transition occurred at $r_g/r_s = 1$, we estimated this transition point analytically. We considered a situation in which only one phenotype exists in the system and is in a steady state and examined whether the invasive phenotype can be fixed. We also consider that nutrient supply events occur at regular intervals; and thus, $\Delta\tau$ is constant ($\Delta\tau_{\text{const.}}$).

First, let $X_i^{(k)}$ be the population of phenotype i at the k-th nutrient supply event ($X_i^{(k)} = X_i(\tau_k)$). The change in population size caused by a nutrient supply event is determined by an increase in population in the feast phase and a decrease in population in the famine phase. Thus, $X_i^{(k+1)}$ can be calculated as

$$X_i^{(k+1)} = X_i^{(k)} \exp\left(\mu_{i,e_k} T_k^+ - \gamma_{i,e_k}(-T_k^+)\right). \tag{4}$$

T_k^+ denotes duration of nutrient availability after k-th nutrient supply event. We define $f_{i,k}$ as the logarithm of the ratio of $X_i^{(k+1)}$ to $X_{i+1}^{(k)}$.

$$f_{i,k} = \mu_{i,e_k} T_k^+ - \gamma_{i,e_k}(\Delta\tau_{\text{const.}} - T_k^+). \tag{5}$$

When $f_{i,k}$ is positive, the population of phenotype i increases, whereas when $f_{i,k}$ is negative, the population decreases. Because the two environments come at random with equal probability, the average value of fitness f_i is

$$f_i = \langle f_{i,k} \rangle = \mu_i T^+ - \gamma_i(\Delta\tau_{\text{const.}} - T^+), \tag{6}$$

where $\mu_i = \dfrac{\mu_{i,A} + \mu_{i,B}}{2}, \gamma_i = \dfrac{\gamma_{i,A} + \gamma_{i,B}}{2}$. Suppose that only one phenotype (phenotype α) exists, and the system is in a steady state; ($X_\alpha^k = X_\alpha^{k+1} = X_\alpha^{st}$), $f_\alpha = 0$ holds.

$$f_\alpha = \mu_\alpha T^+ - \gamma_\alpha(\Delta\tau_{\text{const.}} - T^+) = 0. \tag{7}$$

Then, we consider that a small number of another phenotype (phenotype β) invades the steady state of phenotype α. The number of phenotype β is sufficiently small such that the change in the value of T^+ is negligible. Thus, f_β is given by

$$f_\beta \quad = \mu_\beta T^+ - \gamma_\beta \Delta\tau_{\text{const.}} - T^+). \tag{8}$$

By solving equation Eq (7) for T^+, we get

$$T^+ = \frac{\gamma_\alpha}{\mu_\alpha + \gamma_\alpha} \Delta\tau_{\text{const.}}. \tag{9}$$

Inserting Eq (9) into Eq (8), we get

$$f_\beta = \frac{\Delta\tau_{\text{const.}}}{\mu_\alpha + \gamma_\alpha} \left(\mu_\beta \gamma_\alpha - \mu_\alpha \gamma_\beta\right). \tag{10}$$

Therefore, when $\dfrac{\mu_\beta}{\gamma_\beta} > \dfrac{\mu_\alpha}{\gamma_\alpha}$, f_β becomes positive so that phenotype β can be fixed. On the other hand, when $\dfrac{\mu_\beta}{\gamma_\beta} < \dfrac{\mu_\alpha}{\gamma_\alpha}$, f_β becomes negative and phenotype β is excluded and the steady state with only phenotype α is stable.

3 Evolutionary invasion analysis in $N = 2$ case

For $N = 2$, the generalist is always dominant, and we did not observe a transition between generalist dominance and specialist dominance. To understand this outcome, we analyze invasion fitness in the $N = 2$ setting.

The invasibility of a specialist into a resident generalist population can be discussed in essentially the same way as in the $N = 3$ case. In contrast, the invasibility of a generalist into a resident specialist population requires additional consideration: the $N = 2$ case lacks the A/B symmetry exploited in the $N = 3$ analysis, and therefore the simple averaging argument over the two environments does not apply.

Here, we consider two environments (A, B) and a system in which only a single specialist phenotype is present. Let the specialist's growth rates during feast in A and B be μ_A and μ_B, respectively, and its death rates during famine be γ_A and γ_B, respectively. We assume a constant nutrient supply interval $\Delta\tau$, with nutrients A and B supplied alternately, and that the population dynamics have reached a periodic solution.

Let X_A and X_B denote the specialist population at the moments when nutrient A and nutrient B are supplied, respectively. Then,

$$X_B = X_A \exp(\mu_A T_A^+ - \gamma_A T_A^-), \tag{11}$$

$$X_A = X_B \exp(\mu_B T_B^+ - \gamma_B T_B^-), \tag{12}$$

where T_e^+ and T_e^- are the durations of the feast and famine phases for nutrient e, respectively. Moreover, since $\dot{S} = -\dot{X}$, we have $S(t) = S_0 + X_0 - X(t)$. Substituting $t = T^+$ yields

$$0 = S_0 + X_A - X_A \exp(\mu_A T_A^+), \tag{13}$$

$$0 = S_0 + X_B - X_B \exp(\mu_B T_B^+), \tag{14}$$

Substituting Eqs (13) and (14) into Eqs (11) and (12), we obtain

$$X_B = (S_0 + X_A) \exp(-\gamma_A T_A^-), \tag{15}$$

$$X_A = (S_0 + X_B) \exp(-\gamma_B T_B^-). \tag{16}$$

Therefore,

$$T_A^+ = \frac{1}{\mu_A} \log\left(\frac{S_0 + X_A}{X_A}\right), \tag{17}$$

$$T_A^- = \frac{1}{\gamma_A} \log\left(\frac{S_0 + X_A}{X_B}\right), \tag{18}$$

$$T_B^+ = \frac{1}{\mu_B} \log\left(\frac{S_0 + X_B}{X_B}\right), \tag{19}$$

$$T_B^- = \frac{1}{\gamma_B} \log\left(\frac{S_0 + X_B}{X_A}\right). \tag{20}$$

From Eqs (11), (12) and the identity $T_e^+ + T_e^- = \Delta\tau_{\mathrm{const.}}$ $(e \in A, B)$, we obtain

$$\mu_A T_A^+ - \gamma_A(\Delta\tau_{\mathrm{const.}} - T_A^+) = \log\left(\frac{X_B}{X_A}\right), \tag{21}$$

$$\mu_B T_B^+ - \gamma_B(\Delta\tau_{\mathrm{const.}} - T_B^+) = \log\left(\frac{X_A}{X_B}\right). \tag{22}$$

Substituting Eqs (17) and (19) into Eqs (21) and (22), we obtain

$$\log\left(\frac{S_0 + X_A}{X_B}\right) = \frac{\mu_A \gamma_A}{\mu_A + \gamma_A} \Delta\tau_{\mathrm{const.}}, \tag{23}$$

$$\log\left(\frac{S_0 + X_B}{X_A}\right) = \frac{\mu_B \gamma_B}{\mu_B + \gamma_B} \Delta\tau_{\mathrm{const.}}. \tag{24}$$

Substituting Eqs (13) and (14) into Eqs (18) and (20), we obtain

$$T_A^- = \frac{\mu_A}{\mu_A + \gamma_A} \Delta\tau_{\text{const.}}, \tag{25}$$

$$T_B^- = \frac{\mu_B}{\mu_B + \gamma_B} \Delta\tau_{\text{const.}}. \tag{26}$$

Therefore,

$$T_A^+ = \frac{\gamma_A}{\mu_A + \gamma_A} \Delta\tau_{\text{const.}}, \tag{27}$$

$$T_B^+ = \frac{\gamma_B}{\mu_B + \gamma_B} \Delta\tau_{\text{const.}}. \tag{28}$$

Moreover, since $T_e^+ + T_e^- = \Delta\tau_{\text{const.}}$ $(e \in A, B)$, we have

$$T_A^- = \frac{\mu_A}{\mu_A + \gamma_A} \Delta\tau_{\text{const.}}, \tag{29}$$

$$T_B^- = \frac{\mu_B}{\mu_B + \gamma_B} \Delta\tau_{\text{const.}}. \tag{30}$$

Suppose that a small number of generalists invades this resident specialist population. Let the invading generalist's growth rate be μ_g and its death rate be γ_g. Because the invader is present at a very low abundance, we assume that its impact on the nutrient depletion time is negligible. The net growth rate f of the generalist over the interval between two successive supply events is given by

$$\begin{aligned} f &= \mu_g T_A^+ - \gamma_g T_A^- + \mu_g T_B^+ - \gamma_g T_B^- \\ &= \left(\frac{\mu_g \gamma_A - \gamma_g \mu_A}{\mu_A + \gamma_A} + \frac{\mu_g \gamma_B - \gamma_g \mu_B}{\mu_B + \gamma_B} \right) \Delta\tau_{\text{const.}}. \end{aligned} \tag{31}$$

Fig D shows that the numerically calculated invasion fitness (f) of one strategy when the other is dominant, under the same parameter conditions as the $N = 2$ simulation (S3 Fig in the main text).

The blue line represents the invasion fitness of the generalist against the resident specialist (Eq (31)), while the orange line indicates the invasion fitness of the specialist against the resident generalist (Eq (10)). Throughout the parameter range used in the simulation, the generalist is consistently dominant. In perfect agreement with this simulation result, the analytical invasion fitness of the generalist remains strictly positive ($f > 0$) across this entire range, confirming the consistency between our analytical and numerical results.

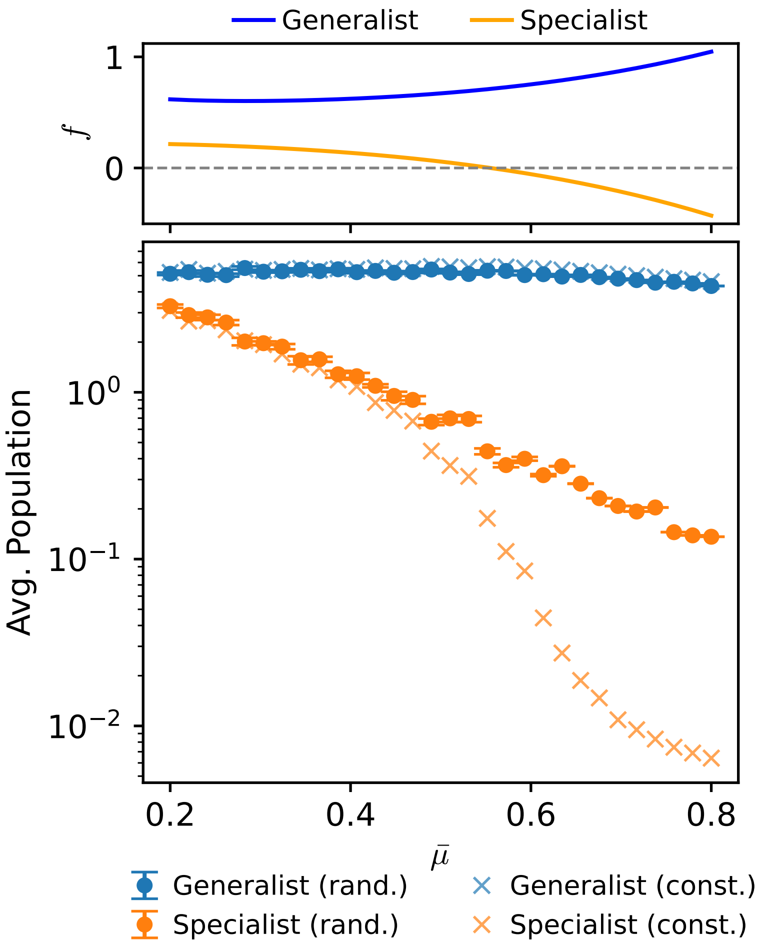

Fig D. Invasion fitness with $N = 2$ case. (Top) Analytical invasion fitness (f) calculated using the parameter values from the corresponding simulations. The blue line represents the invasion fitness of the generalist against a resident specialist population, while the orange line indicates the invasion fitness of the specialist against a resident generalist population. Across the entire parameter range, the generalist maintains a strictly positive invasion fitness ($f > 0$), indicating its ability to invade and dominate. (Bottom) Numerical simulation results under the same parameter conditions as shown in S3 Fig.

4 Analytical solution for the temporally averaged population under generalist-dominant condition

To explain the increase in specialists under generalist-dominant conditions, we analytically estimated the temporally averaged population using a simplified model under three assumptions.

1. Specialist populations are small enough that phenotypic change from specialist to generalist is negligible.

2. Two environments A and B change alternately and $\Delta\tau$ is constant ($\Delta\tau_{\text{const.}}$).

3. The dynamics settle into a periodic solution. The generalist population shows no net change between the nutrient supply events. Specialist populations show no net change between the two consecutive supply events.

There are three phenotypes (phenotype 1, 2 and 3) and two environmental conditions (environment A and B). Phenotype 1 is a specialist in environment A, phenotype 3 is a specialist in environment B, and phenotype 2 is a generalist. We assume that the two environments A and B are symmetric. Thus, we set the growth rates as follows using the three variables μ_s^+, μ_s^-, and μ_g ($\mu_s^- < \mu_g < \mu_s^+$),

$$\mu_{1,A} = \mu_{3,B} = \mu_s^+,\ \mu_{1,B} = \mu_{3,A} = \mu_s^-,\ \mu_{2,A} = \mu_{2,B} = \mu_g. \tag{32}$$

Similarly, we set the death rates as

$$\gamma_{1,A} = \gamma_{3,B} = \gamma_s^+,\ \gamma_{1,B} = \gamma_{3,A} = \gamma_s^-,\ \gamma_{2,A} = \gamma_{2,B} = \gamma_g, \tag{33}$$

where $\gamma_s^- < \gamma_g < \gamma_s^+$ holds true. Phenotype 2 grows at a rate μ_g and switches to phenotypes 1 and 3 at a rate p, respectively. Therefore, generalist population X_2 is as follows:

$$\frac{dX_2}{dt} = \begin{cases} (1-2p)\mu_g X_2 & (\tau_k < t < \tau_k + T_k^+) \\ -\gamma_g X_2 & (\tau_k + T_k^+ < t < \tau_{k+1}) \end{cases}, \tag{34}$$

where T_k^+ is the duration of nutrient availability after the k-th nutrient supply event. The specialist population increases through the sum of its intrinsic growth and inflow of individuals from the generalist population. When the k-th nutrient supply event places the system in environment e, specialist populations X_1 and X_3 follow the following equations:

$$\frac{dX_i}{dt} = \begin{cases} \mu_{i,e} X_i + p\mu_g X_2 & (\tau_k < t < \tau_k + T_k^+) \\ -\gamma_{i,e} X_i & (\tau_k + T_k^+ < t < \tau_{k+1}) \end{cases}. \tag{35}$$

Because each supply event resets the nutrient concentration to a fixed level S_0, which is subsequently depleted by the three phenotypes, the nutrient dynamics are governed by the following equation.

$$S(\tau_k) = S_0,\ \frac{dS}{dt} = -\left(\mu_g X_2 + \mu_{1,e} X_1 + \mu_{3,e} X_3\right) \quad (\tau_k < t < \tau_k + T_k^+). \tag{36}$$

First, we calculated the temporally averaged population of the generalist. By solving Eq (34), we obtain:

$$X_2(\tau_k + t') = \begin{cases} X_2(\tau_k)\exp(\mu_g^* t') & (0 < t' < T_k^+) \\ X_2(\tau_k)\exp(\mu_g^* T_k^+ - \gamma_g(t' - T_k^+)) & (T_k^+ < t' < \Delta\tau_{\text{const.}}) \end{cases}, \tag{37}$$

where $\mu_g^* = (1-2p)\mu_g$ is the effective growth rate of the generalist.

Assuming that there is no change in the population between nutrient supply events, we have

$$X_2(\tau_k) = X_2(\tau_{k+1}) = X_g^*.$$

Accordingly, the condition

$$\exp(\mu_g^* T_k^+ - \gamma_g(\Delta\tau_{\text{const.}} - T_k^+)) = 1 \tag{38}$$

holds. Thus, T_k^+ is given by

$$T_k^+ = \frac{\gamma_g}{\mu_g^* + \gamma_g}\Delta\tau_{\text{const.}}. \tag{39}$$

By integrating X_2 from τ_k to τ_{k+1}, we can calculate the temporally averaged population of the generalist from τ_k to τ_{k+1}, $\langle X_2 \rangle_k$:

$$\begin{aligned}\langle X_2\rangle_k &= \frac{1}{\Delta\tau_{\text{const.}}}\left(\int_{\tau_k}^{\tau_k+T_k^+} X_2(t)\,dt + \int_{\tau_k+T_k^+}^{\tau_{k+1}} X_2(t)\,dt\right)\\ &= X_g^*\left(\frac{1}{\mu_g^*}+\frac{1}{\gamma_g}\right)\left(\exp(\mu_g^* T_k^+) - 1\right),\end{aligned} \tag{40}$$

where we utilized Eq (38) to obtain the final equality.

Next, we calculated the population of specialists. Because we assumed that environment A and B are symmetric and change alternately, the periodic solution of the specialist populations X_1 and X_3 are symmetric with respect to the two environmental conditions. That is, $X_1(t'+\tau_k) = X_3(t'+\tau_{k+1})$ and $X_3(t'+\tau_k) = X_1(t'+\tau_{k+1})$ hold for all t' satisfying $0 < t' < \Delta\tau_{\text{const.}}$. Therefore, it is sufficient to solve solely for X_1.

To simplify the calculations, we introduced the dimensionless quantity $R = X_1/X_2$. Because $\dot{R} = \frac{\dot{X}_1}{X_2} - R\frac{\dot{X}_2}{X_2}$, it follows from Eq (34) and Eq (35) that

$$\frac{dR}{dt} = \begin{cases} \left(\mu_{1,e} - \mu_g^*\right)R + p\mu_g & (\tau_k < t < \tau_k + T_k^+),\\ -\left(\gamma_{1,e} - \gamma_g\right)R & (\tau_k + T_k^+ < t < \tau_{k+1}).\end{cases} \tag{41}$$

By solving Eq (41), we obtain

$$R(\tau_k + t') = \begin{cases} R(\tau_k)\exp(\Delta\mu_e t') + \frac{p\mu_g}{\Delta\mu_e}\left(\exp(\mu_e t') - 1\right) & (0 < t' < T_k^+),\\ R(\tau_k + T_k^+)\exp\left(-\Delta\gamma_e(t' - T_k^+)\right) & (T_k^+ < t' < \Delta\tau_{\text{const.}}),\end{cases} \tag{42}$$

where $\Delta\mu_e = \mu_{1,e} - \mu_g^*$ and $\Delta\gamma_e = \gamma_{1,e} - \gamma_g$.

Assuming that the dynamics settle into a periodic orbit, the population of phenotype 1 returns to its original value after experiencing environment A and B. Therefore, $R(\tau_k) = R(\tau_{k+2})$ holds. In the following, we now solve for $R(\tau_k)$ satisfying this equation.

We introduced the discrete map between $R(\tau_k)$ and $R(\tau_{k+1})$, F_e where $F_e(R(\tau_k)) = R(\tau_{k+1})$ holds. From Eq (42), F_e can be expressed as a linear function of R

$$F_e(R(\tau_k)) = E_e R(\tau_k) + D_e, \tag{43}$$

where $E_e = \exp\left(\Delta\mu_e T_k^+ - \Delta\gamma_e(\Delta\tau_{\text{const.}} - T_k^+)\right)$ and $D_e = \frac{p\mu_g}{\Delta\mu_e}\left(E_e - \exp(-\Delta\gamma_e(\Delta\tau_{\text{const.}} - T_k^+))\right)$.

When the k-th environment is A, the $(k+1)$-th environment is B, and $R(\tau_{k+2})$ can be calculated as:

$$R(\tau_{k+2}) = F_B(F_A(R(\tau_k))) = E_A E_B R(\tau_k) + D_A E_B + D_B. \tag{44}$$

By setting $R(\tau_k) = R(\tau_{k+2}) = R_A^*$, we obtain

$$R_A^* = \frac{D_A E_B + D_B}{1 - E_A E_B}. \tag{45}$$

Analogously, when the k-th environment is B, we can obtain R_B^* by the same procedure as in Eq (44) and Eq (45).

$$R_B^* = \frac{D_B E_A + D_A}{1 - E_A E_B}. \tag{46}$$

Assuming that the system has settled into a periodic solution, Eq (42) can be reformulated using Eq (45) and Eq (46), as follows:

$$R(\tau_k + t') = \begin{cases} R_e^* \exp(\Delta\mu_e t') + \frac{p\mu_g}{\Delta\mu_e}\left(\exp(\Delta\mu_e t') - 1\right) & (0 < t' < T_k^+), \\ R_e^*(T_k^+) \exp(-\Delta\gamma_e (t' - T_k^+)) & (T_k^+ < t' < \Delta\tau_{\mathrm{const.}}), \end{cases} \tag{47}$$

where $R_e^*(T_k^+) = R_e^* \exp(\Delta\mu_e t') + \frac{p\mu_g}{\Delta\mu_e}\left(\exp(\Delta\mu_e t') - 1\right)$.

The temporally averaged population of phenotype 1 from $t = \tau_k$ to τ_{k+1} is obtained by integrating RX_2 from $t = \tau_k$ to τ_{k+1} in each environment e, and then averaging the values over the two environments, that is

$$\langle X_1 \rangle_k = \frac{1}{2\Delta\tau_{\mathrm{const.}}} \sum_{e=A,B} \left(\int_{\tau_k}^{\tau_k + T_k^+} R(t; e_k = e) X_g(t)\, dt + \int_{\tau_k + T_k^+}^{\tau_{k+1}} R(t; e_k = e) X_g(t)\, dt \right). \tag{48}$$

The first and the second terms of the integral are given by

$$\begin{aligned} \int_{\tau_k}^{\tau_k + T_k^+} R(t; e_k = e) X_g(t)\, dt = {} & \frac{X_g^* R_e^*}{\mu_{1,e}} \left(\exp(\mu_{1,e} T_k^+) - 1 \right) \\ & + \frac{X_g^* p\mu_g}{\Delta\mu_e} \left(\frac{\exp(\mu_{1,e} T_k^+) - 1}{\mu_{1,e}} - \frac{\exp(\mu_g^* T_k^+) - 1}{\mu_g^*} \right), \end{aligned} \tag{49}$$

and

$$\int_{\tau_k + T_k^+}^{\tau_{k+1}} R(t; e_k = e) X_g(t)\, dt = X_g^* \exp(\mu_g^* T_k^+) R_e^*(T_k^+) \frac{1 - \exp(-\gamma_{1,e}(\Delta\tau_{\mathrm{const.}} - T_k^+))}{\gamma_{1,e}}, \tag{50}$$

respectively.

By inserting Eq (49) and Eq (50) into Eq (48), we can obtain

$$\langle X_1 \rangle_k = \frac{X_g^*}{2\Delta\tau_{\mathrm{const.}}} \sum_{e=A,B} (\mathcal{R}_e + \mathcal{S}_e), \tag{51}$$

where

$$\mathcal{R}_e = \frac{R_e^*}{\mu_{1,e}} \left(\exp(\mu_{1,e} T_k^+) - 1 \right) + \frac{p\mu_g}{\Delta\mu_e} \left(\frac{\exp(\mu_{1,e} T_k^+) - 1}{\mu_{1,e}} - \frac{\exp(\mu_g^* T_k^+) - 1}{\mu_g^*} \right) \tag{52}$$

$$\mathcal{S}_e = \exp(\mu_g^* T_k^+) R_e^*(T_k^+) \frac{1 - \exp(-\gamma_{1,e}(\Delta\tau_{\mathrm{const.}} - T_k^+))}{\gamma_{1,e}}. \tag{53}$$

The temporally averaged populations of generalists and specialists are given by Eq (40) and Eq (51), respectively: however, constant X_g^* remains undetermined. In the following, we calculate X_g^* using Eq (36).

Because the periodic solutions of the specialist populations are symmetric with respect to environment A and B, $\mu_{3,A}X_3 = \mu_{1,B}X_1$ and $\mu_{1,A}X_1 = \mu_{3,B}X_3$ hold true.

Therefore, Eq (36) can be rewritten as

$$\frac{dS}{dt} = -\mu_g X_2 - \mu_{1,A}X_1 - \mu_{1,B}X_1. \tag{54}$$

We integrate Eq (54) from τ_k to $\tau_k + T_k^+$.

$$\begin{aligned} S_0 &= \mu_g \int_{\tau_k}^{\tau_k+T_k^+} X_2(t)\,dt + \sum_{e=A,B} \mu_{1,e} \int_{\tau_k}^{\tau_k+T_k^+} R(t; e_k = e) X_2(t)\,dt \\ &= \frac{X_g^* \mu_g}{\mu_g^*} \left(\exp(\mu_g^* T_k^+) - 1\right) + \sum_{e=A,B} \mu_{1,e} X_g^* \mathcal{R}_e. \end{aligned} \tag{55}$$

By solving Eq (55) for X_g^*, we obtain

$$X_g^* = \frac{S_0}{\frac{\mu_g}{\mu_g^*}\left(\exp(\mu_g^* T_k^+) - 1\right) + \sum_{e=A,B} \mu_{1,e}\mathcal{R}_e}. \tag{56}$$

Combining Eq (40) and Eq (51) and Eq (56), we obtain the temporally averaged population of generalists and specialists.

$$\begin{aligned} X_2^{\text{approx}}(\Delta\tau_{\text{const.}}) &= \frac{\sum_k \langle X_2 \rangle_k \Delta\tau_{\text{const.}}}{\sum_k \Delta\tau_{\text{const.}}} \\ &= \frac{1}{\Delta\tau_{\text{const.}}} \frac{S_0\left(\exp\left(\mu_g^* T_k^+\right) - 1\right)}{\frac{\mu_g}{\mu_g^*}(\exp\left(\mu_g^* T_k^+\right) - 1) + \sum_{e=A,B} \mu_{1,e}\mathcal{R}_e} \left(\frac{1}{\mu_g^*} + \frac{1}{\gamma_g}\right), \end{aligned} \tag{57}$$

$$\begin{aligned} X_1^{\text{approx}}(\Delta\tau_{\text{const.}}) &= \frac{\sum_k \langle X_1 \rangle_k \Delta\tau_{\text{const.}}}{\sum_k \Delta\tau_{\text{const.}}} \\ &= \frac{1}{\Delta\tau_{\text{const.}}} \frac{S_0 \sum_{e=A,B} \left(\mathcal{R}_e + \mathcal{S}_e\right)}{\frac{\mu_g}{\mu_g^*}(\exp\left(\mu_g^* T_k^+\right) - 1) + \sum_{e=A,B} \mu_{1,e}\mathcal{R}_e}, \end{aligned} \tag{58}$$

where T_k^+,$\mathcal{R}_e$ and $\mathcal{S}_e$ are defined by Eq (39), Eq (52) and Eq (53), respectively.

Fig E shows a comparison of the analytical solution with the numerical simulation results as a function of $\Delta\tau$. The simulation parameters are identical to those used in Fig 4B in the main text (e.g. $\bar{\mu} = 0.8$). We substituted these parameter values into Eq (57) and Eq (58) to compute the corresponding analytical solutions. The dots indicate the simulation results, and the dashed curves show the analytical solution. Fig E(a) shows simulations in which environments A and B alternate deterministically, while Fig E(b) shows simulations in which the sequence of environments A and B is randomized. In simulations with alternating environments, the analytical solution was in good agreement with the simulation results. In simulations with randomly fluctuating environments, the analytical prediction for the generalist deviates at a large $\Delta\tau$; nonetheless, it still captures the principal qualitative trend.

Fig F compares the numerical simulations and analytical predictions as a function of $\bar{\mu}$. Panels (a) and (b) correspond to deterministic alternation of environments A and B and randomized environmental sequences, respectively. Because the analytical expression assumes that the generalist remains dominant, we plotted the analytical curves exclusively within the generalist-dominant regime, omitting them where this assumption fails. Within this applicable range, the agreement is essentially exact under deterministic alternation, and the analytical curve captures the overall trends well under random switching.

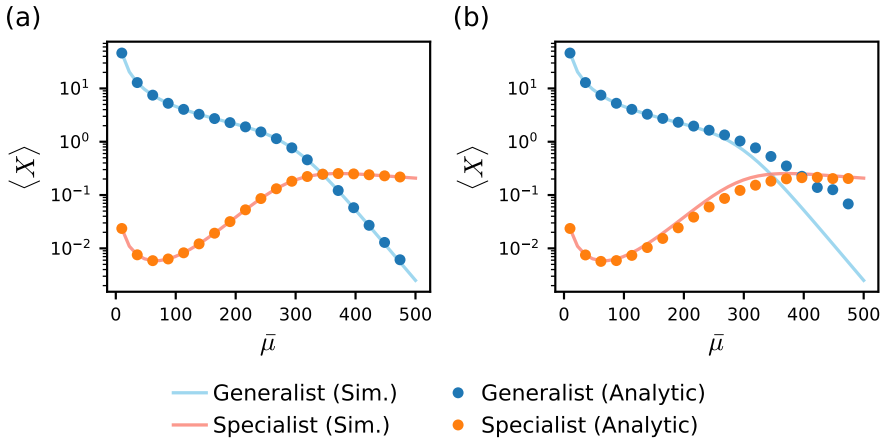

Fig E. Comparison of the analytical solution with the numerical simulation results as a function of $\Delta\tau$. The dots indicate the simulation results, and the dashed curves show the analytical solution. (a) Simulations in which environments A and B alternate deterministically. (b) Simulations in which the sequence of environments A and B is randomized. The nutrient supply interval $\Delta\tau$ is kept constant across simulations. The parameters are set to $\mu_{2,A} = \mu_{2,B} = 0.8$, $\mu_{1,A} = \mu_{3,B} = 1.6$, $\mu_{1,B} = \mu_{3,A} = 0.4$, $a = 0.01$, $b = 1$, $p = 10^{-4}$ and $\theta = 10^{-8}$.

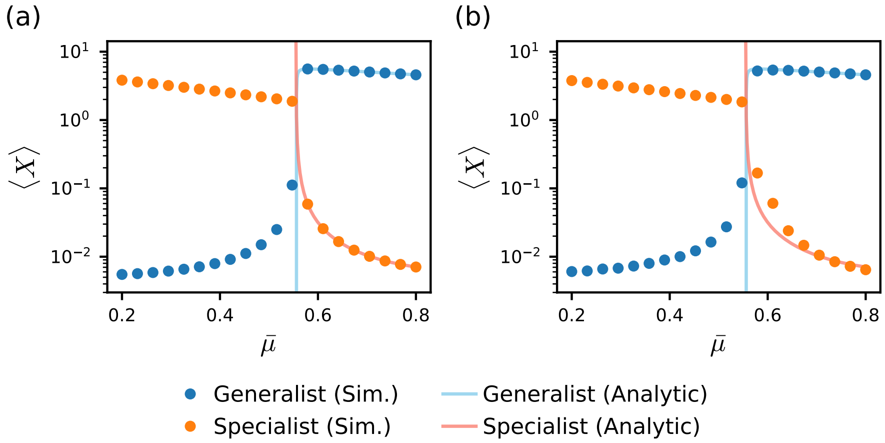

Fig F. Comparison of the analytical solution with the numerical simulation results as a function of $\bar{\mu}$. The dots indicate the simulation results, and the dashed curves show the analytical solution. (a) Simulations in which environments A and B alternate deterministically. (b) Simulations in which the sequence of environments A and B is randomized. The nutrient supply interval is kept constant ($\Delta\tau_{\text{const.}} = 100$). The parameters are set to $\mu_{2,A} = \mu_{2,B} = \bar{\mu}$, $\mu_{1,A} = \mu_{3,B} = 2\bar{\mu}$, $\mu_{1,B} = \mu_{3,A} = \bar{\mu}/2$, $a = 0.01$, $b = 1$, $p = 10^{-4}$ and $\theta = 10^{-8}$.

5 Consideration of Multiple Nutrients

In this study, we simplified the model under the assumption that at most one type of nutrient exists in the system at any given time. However, in actual natural environments, multiple nutrients often coexist. Here, we demonstrate that the main qualitative conclusions obtained in the main text remain unchanged even in a model setting that considers the case where multiple nutrients are supplied simultaneously.

Similar to the model in the main text, each phenotype i has a growth parameter $\mu_{i,e}$ for each nutrient e. We assume that the growth rate μ_i of phenotype i is given by the sum of the growth parameters for the available nutrients. That is,

$$\mu_i = \sum_{e=1}^{E} \mu_{i,e} \mathbb{1}(S_e > 0), \quad (59)$$

where $\mathbb{1}$ is the indicator function.

When all nutrients are depleted, the population size begins to decrease. Let γ_i be the death rate, and we consider the growth-death trade-off as follows:

$$\gamma_i = a \exp\left(b \sum_{e=1}^{E} \mu_{i,e} \right). \quad (60)$$

As in the main text, we consider two environments (A, B) and three phenotypes (1, 2, 3). We set the growth rates as in Fig 2B in the main text. In the nutrient supply event, nutrients A and B are supplied simultaneously in equal amounts. Fig G shows the time-averaged population size of each phenotype as a function of $\bar{\mu}$.

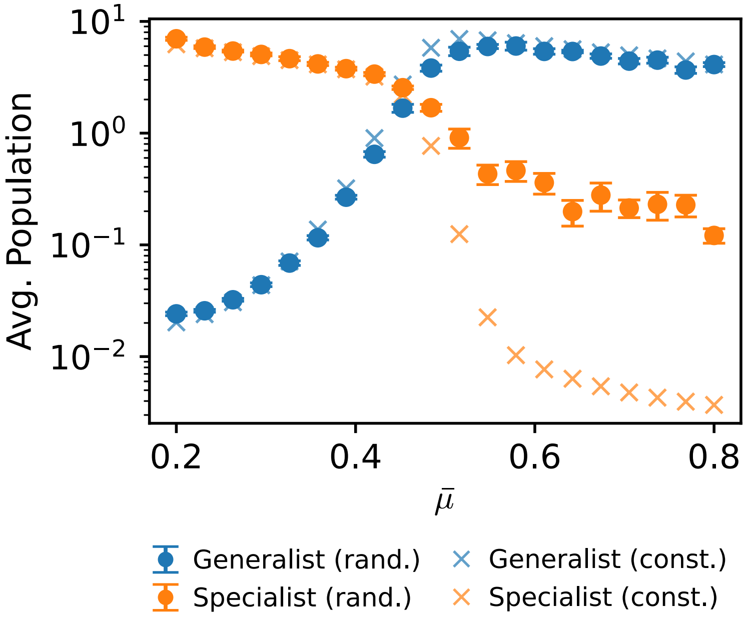

Fig G. Temporal average of the population under multiple types of nutrients. The temporally averaged populations of the one generalist and two specialists are shown as a function of the parameter $\bar{\mu}$. Crosses ($\times$) denote results with constant nutrient supply intervals ($\Delta\tau_k = 100$), whereas circles ($\bullet$) denote those with gamma-distributed intervals ($\Delta\tau_k \sim \Gamma(2, 50)$). At each nutrient supply event, both A and B are supplied simultaneously, each with an amount S_0. We simulated up to $t = 3.0 \times 10^5$ and averaged the populations after $t = 2.5 \times 10^5$. For each $\bar{\mu}$, we ran 10 simulations and averaged the results. The error bars indicate the standard error. All other parameters were identical to those used in Fig 3 in the main text.

6 Consideration of Concentration-dependent Growth Rates

In the main model, we assumed that the growth rate is constant as long as nutrients are present. However, it is known that the actual growth rate of microorganisms depends on nutrient concentration. For example, the Monod equation, expressed below, is widely used as a standard model for the specific growth rate on a single nutrient [2].

$$\mu(S) = \mu_{\max} \frac{S}{S+K}, \tag{61}$$

In the following, we demonstrate that the main qualitative conclusions obtained in the main text remain unchanged even when the growth rate decreases monotonically with respect to the nutrient concentration.

First, we extend the model from the main text to simulate the case where the growth rate depends on the nutrient concentration S. The growth rate of each phenotype is given as follows:

$$\mu_{i,e}(S) = \mu_{i,e}^{\max} \frac{S}{S+K}. \tag{62}$$

Here, we assume that K is common to all phenotypes.

We consider two environments (A, B) and three phenotypes (1, 2, 3), and assign $\mu_{i,e}^{\max}$ as follows:

$$\begin{aligned} \mu_{1,A}^{\max} = 2\mu_{\max},\ \mu_{1,B}^{\max} = \mu_{\max}/2, \\ \mu_{2,A}^{\max} = \mu_{2,B}^{\max} = \mu_{\max}, \\ \mu_{3,A}^{\max} = \mu_{\max}/2,\ \mu_{3,B}^{\max} = 2\mu_{\max}. \end{aligned} \tag{63}$$

We assume that the death rate during the famine phase increases depending on $\mu_{i,e}^{\max}$, given by:

$$\gamma_{i,e} = a \exp\left(b\mu_{i,e}^{\max}\right). \tag{64}$$

When the growth rate follows the Monod equation, the rate of nutrient consumption decreases as nutrients decrease; thus, nutrients are never completely depleted. Here, to represent growth during the feast phase and decline during the famine phase, we introduce a cellular nutrient detection threshold S_{th} and consider the system to be in the famine phase if the nutrient concentration satisfies $S < S_{\text{th}}$.

We performed simulations by systematically varying the value of $\mu_{\max}$, and the results of the time-averaged population size for each phenotype are shown in Fig H. A transition between specialist dominance and generalist dominance was observed similarly to the main text.

Next, we consider invasion analysis similar to Supplementary text Section 2. Unless otherwise noted, variables are defined as in Section 2. Let the growth rate of phenotype i be expressed as $\mu_i^{\max} f(S)$ using a function $f(S)$ that depends on the nutrient concentration S (the case $f(S) = \frac{S}{S+K}$ corresponds to the Monod equation). First, assume that only phenotype α exists and the system is in a steady state on a time-averaged basis. In this case, f_α is:

$$f_\alpha = \mu_\alpha^{\max} \int_0^{T^+} f(S)\, dt - \gamma_\alpha(\Delta\tau - T^+) = 0. \tag{65}$$

Next, consider the case where another phenotype β invades in small numbers into this steady state of phenotype α alone. We assume that the population size of the

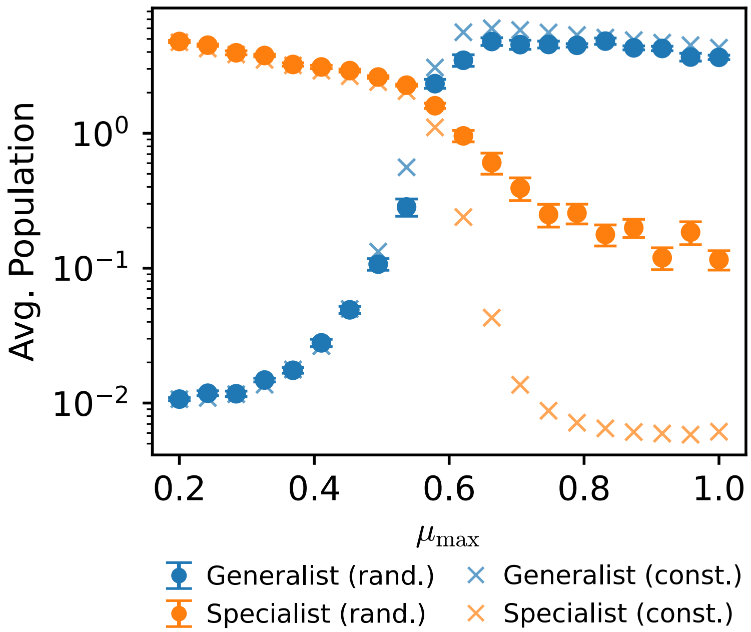

Fig H. Temporal average of the population under concentration-dependent growth. The temporally averaged populations of the one generalist and two specialists are shown as a function of the parameter $\mu_{\max}$. Crosses ($\times$) denote results with constant nutrient supply intervals ($\Delta\tau_k = 100$), whereas circles ($\bullet$) denote those with gamma-distributed intervals ($\Delta\tau_k \sim \Gamma(2, 50)$). At each nutrient supply event, At each nutrient supply event, one of the two nutrient types (A or B) is chosen at random. We simulated up to $t = 3.0 \times 10^5$ and averaged the populations after $t = 2.5 \times 10^5$. For each $\mu_{\max}$, we ran 10 simulations and averaged the results. The error bars indicate the standard error. All other parameters were identical to those used in Fig 3 in the main text.

invading phenotype β is sufficiently small so that the accompanying change in the dynamics of S is negligible. In this case, f_β is given by:

$$f_\beta = \mu_\beta^{\max} \int_0^{T^+} f(S)\, dt - \gamma_\beta(\Delta\tau - T^+). \tag{66}$$

From Eqs (65) and (66), we obtain:

$$f_\beta = \frac{1}{\gamma_\alpha} \left(\gamma_\alpha \mu_\beta^{\max} - \gamma_\beta \mu_\alpha^{\max}\right) \int_0^{T^+} f(S)\, dt. \tag{67}$$

Here, if we define

$$\mu_i^* = \frac{1}{T^+} \mu_i^{\max} \int_0^{T^+} f(S)\, dt. \tag{68}$$

as the mean growth rate of phenotype i during the feast phase, Eq (67) can be written as:

$$f_\beta = \frac{T^+}{\gamma_\alpha} \left(\gamma_\alpha \mu_\beta^* - \gamma_\beta \mu_\alpha^*\right). \tag{69}$$

Therefore, if the ratio of the mean growth rate to the mean death rate (μ_i^*/γ_i) is higher for phenotype β, then $f_\beta > 0$; if it is lower, then $f_\beta < 0$. This indicates that regardless of the specific functional form $f(S)$ of the nutrient concentration dependence of the growth rate, the invasibility is determined by the ratio of the mean growth rate to the mean death rate.

7 Dependence of results on the growth-death trade-off function

In this section, we investigate the influence of the shape of the growth-death trade-off on the results. Here, we assume the following relationship between the growth rate μ and the death rate γ of phenotype i in environment e:

$$\gamma_{i,e} = a + b\mu_{i,e}^{\alpha} \tag{70}$$

Figs I(a) and (b) show the temporally averaged populations for sublinear trade-off ($\alpha = 0.8$) and linear trade-off ($\alpha = 1.0$), respectively.

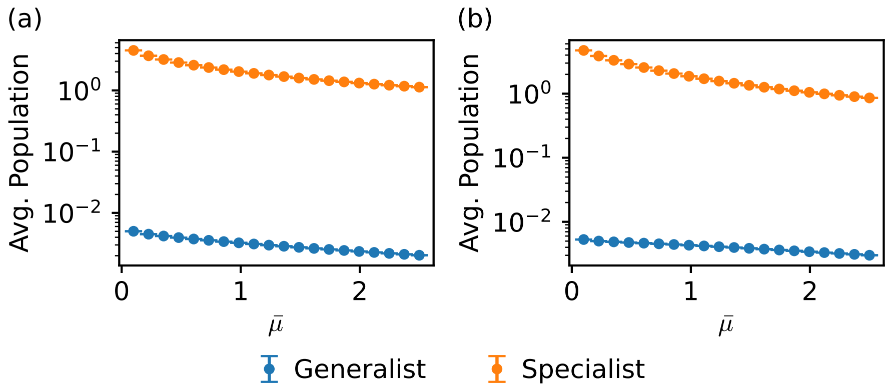

Fig I. Temporal average of the population for sublinear/linear growth-death trade-off. The temporally averaged populations are shown as a function of the parameter $\bar{\mu}$ for (a) the sublinear ($\alpha = 0.8$) and (b) linear ($\alpha = 1.0$) growth-death trade-off. The model with one generalist and two specialists is simulated. We simulated up to $t = 3.0 \times 10^5$ and averaged the populations after $t = 2.5 \times 10^5$. For each $\bar{\mu}$, we ran 10 simulations and averaged the results. The error bars indicate the standard error. The parameters are set to $a = b = 0.01$. All other parameters were identical to those used in Fig 3 in the main text.

The simulations suggest that generalist phenotype cannot be dominant under a sublinear or linear growth-death trade-off. In the following, we demonstrate this analytically.

We consider two environmental conditions and denote the growth rates in these environments by x and y, respectively. We make the following assumptions:

1. The resource-use trade-off function $y = \phi(x)$ is convex.

2. The resource-use trade-off is symmetric with respect to the two environments.

The result in the main text concluded that phenotypes with a higher growth-to-death ratio become dominant in the population. Given this, we show that the growth-to-death ratio takes its minimum value at the generalist phenotype.

First, we consider the case of $\alpha < 1$, namely, the case of a sublinear growth-death trade-off. We define f as the ratio of the arithmetic mean of growth rates to the arithmetic mean of death rates.

$$f = \frac{x + \phi(x)}{2a + b(x^{\alpha} + \phi^{\alpha}(x))}. \tag{71}$$

Let g be the reciprocal of f, $g = f^{-1}$

$$g = \frac{2a}{x+\phi(x)} + \frac{x^\alpha + \phi^\alpha(x)}{x+\phi(x)}. \tag{72}$$

Due to the convexity and symmetry of ϕ, $x+\phi(x)$ reaches its minimum at $x = \phi(x)$. Therefore, the first term of Eq (72) takes its maximum value at $x = \phi(x)$.

We now turn our attention to the second term of Eq (72). Given that t^α is concave for $\alpha < 1$, Jensen's inequality yields:

$$\frac{x^\alpha + \phi^\alpha(x)}{2} \leq \left(\frac{x+\phi(x)}{2}\right)^\alpha, \tag{73}$$

where equality holds if and only if $x = \phi(x)$. We obtain the following inequality:

$$\frac{x^\alpha + \phi^\alpha(x)}{x+\phi(x)} \leq 2^{1-\alpha} \left(x+\phi(x)\right)^{\alpha-1}, \tag{74}$$

Since $\alpha < 1$, we have $\alpha - 1 < 0$. Consequently, the right-hand side of Eq (74) is maximized when $x+\phi(x)$ is minimized, which occurs at $x = \phi(x)$. Furthermore, since the equality holds when $x = \phi(x)$, the left-hand side also attains its maximum value at $x = \phi(x)$. Given that both the first and second terms of g are maximized at $x = \phi(x)$, the function g itself takes its maximum value at this point. Recalling that f is the reciprocal of g, we conclude that f attains its minimum value at $x = \phi(x)$.

Therefore, under a sublinear growth-death trade-off, the generalist phenotype does not emerge as a dominant strategy; instead, specialists are always favored.

Similarly, for a linear growth-death trade-off, we can demonstrate that the growth-death ratio of the generalist phenotype is minimized. Substituting $\alpha = 1$ into Eq (72), the second term becomes a constant. Therefore, as in the sublinear case, f takes its minimum value at $x = \phi(x)$.

Taken together, we showed that the specialist phenotype is invariably favored under sublinear and linear functions. On the other hand, this argument does not hold under a supralinear trade-off. In this regime, a transition between generalist and specialist phenotypes may occur.

Indeed, numerical simulations confirmed a transition in dominance between these strategies. Fig J shows the temporally averaged population for supralinear trade-off ($\alpha = 1.2$).

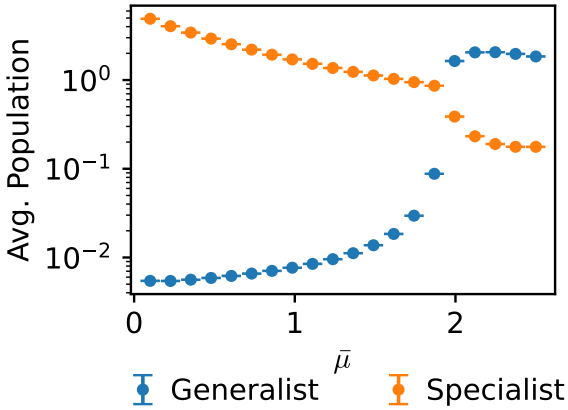

Fig J. Temporal average of the population for sublinear/linear growth-death trade-off. The temporally averaged populations are shown as a function of the parameter $\bar{\mu}$ for supralinear trade-off ($\alpha = 1.2$). Other simulation settings are the same as in Fig I.

8 Simulation with asymmetrically sampled environments

In the main text, we assumed that two environments (A and B) are sampled with equal probabilities. To investigate the robustness of our findings against asymmetric environmental frequencies, we analyzed a scenario where the two environments are selected with asymmetric probabilities.

We denote the probabilities of encountering Environment A and Environment B at each nutrient supply event as P_A and P_B, respectively ($P_A + P_B = 1$). Because the model parameters for the two environments are symmetric, we focused on scenarios where Environment B is sampled more frequently ($P_B \geq 0.5$). As shown in Fig K, as the probability of Environment B increases (from $P_B = 0.5$ to 1.0), the specialist adapted to this environment (Specialist 2) becomes increasingly dominant, whereas the population of Specialist 1 decreases.

Interestingly, however, even when Specialist 1's preferred environment is rarely sampled (e.g., $P_A = 0.1$ and $P_B = 0.9$), there still exists a parameter region where Specialist 1 outperforms the generalist, provided that P_A is non-zero. Specialist 1 faces extinction only when Environment A completely disappears ($P_A = 0.0$); otherwise, it manages to maintain a higher abundance than the generalist in specific parameter regimes.

This result indicates that the competitive advantage of specialists relies on the existence of complementary partners; by functionally complementing each other across the fluctuating environments, the specialists can collectively outcompete the generalist, even when environmental frequencies are highly asymmetric.

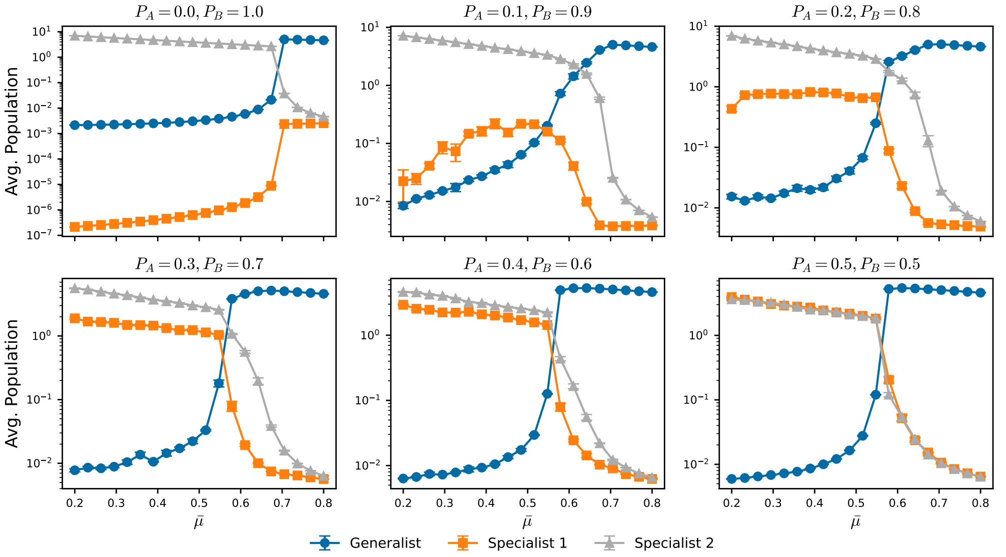

Fig K. Simulation with asymmetrically sampled environments. Temporally averaged populations as a function of $\bar{\mu}$ when two environments are selected with asymmetrical probabilities. Specialist 1 is adapted to environment A, whereas Specialist 2 is adapted to environment B. The interval between nutrient supply events is constant ($\Delta\tau_{\text{const.}} = 100$). At each supply event, environment A or B is randomly selected with probabilities P_A and P_B, respectively, as specified in the title of each panel. All other parameters are identical to those in Fig 3 of the main text.

9 Population dynamics with an asymmetrically specialized generalist

In the main text, we primarily focused on a perfectly symmetric generalist, whose growth rates are identical in both environments ($\mu_A = \mu_B$). To investigate the effect of a generalist having a slight specialization toward a specific environment, we analyzed the population dynamics under scenarios where the generalist's growth rates are asymmetrically biased.

As shown in Fig L, we performed simulations where the generalist has a slight growth advantage in Environment A compared to Environment B (e.g., $\mu_{g,A} = 1.1\bar{\mu}$, $\mu_{g,B} = \bar{\mu}/1.1$). Meanwhile, the specific growth rates of the two specialists are fixed at $(2\bar{\mu}, \bar{\mu}/2)$ for Specialist 1 and $(\bar{\mu}/2, 2\bar{\mu})$ for Specialist 2.

Consistent with our main results, we still observe a clear transition between generalist dominance and specialist dominance depending on the baseline growth rate $\bar{\mu}$. Under specialist-dominant conditions (at lower $\bar{\mu}$), the generalist population is heavily suppressed, and we observe no significant difference in the abundances of Specialist 1 and Specialist 2.

However, under generalist-dominant conditions (at higher $\bar{\mu}$), the dynamics of the two specialists significantly diverge. Because the generalist's growth is biased toward Environment A, it exhibits a higher niche overlap with Specialist 1 than with Specialist 2. This intensified competition strongly suppresses the population of Specialist 1. Conversely, Specialist 2 benefits from a reduced niche overlap with the generalist, allowing it to maintain a relatively higher abundance even when the generalist dominates the community.

These results demonstrate that while a slight specialization of the generalist does not alter the fundamental condition for the generalist-specialist transition, it introduces asymmetric competition within the community. The resulting dynamics highlight how nuanced niche overlaps can drive the differential survival and suppression of specific specialist phenotypes.

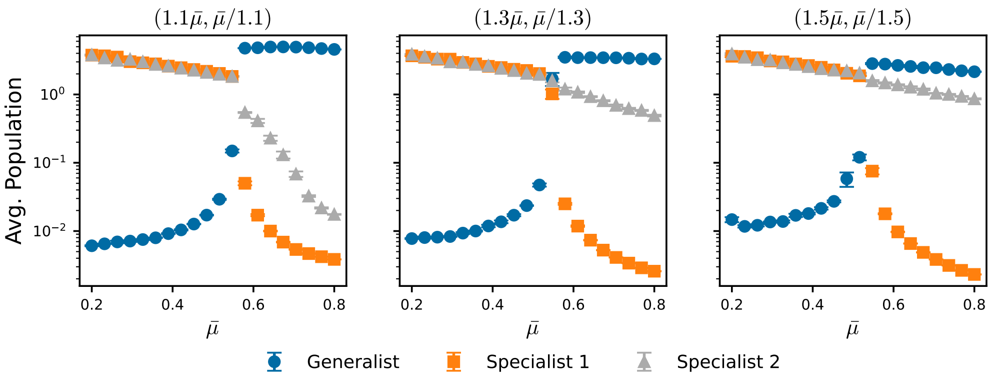

Fig L. Population dynamics with an asymmetrically specialized generalist. Temporally averaged population of the generalist and two specialists as a function of $\bar{\mu}$. In these simulations, the generalist's growth rates are slightly biased toward environment A. The title of each panel indicates the generalist's growth rates in the two environments. Specialist 1 is adapted to environment A, whereas Specialist 2 is adapted to environment B. All other parameters are identical to those in Fig 3 in the main text.

10 Robustness of the generalist-specialist transition against changes in the trade-off shape

In the main text, we primarily evaluated the community dynamics by assuming that all phenotypes lie on a specific growth-death trade-off boundary. To investigate the robustness of our findings against changes in the shape of this trade-off, we analyzed a scenario where the phenotypes of the two specialists are fixed, while the generalist's growth rate is varied independently along the symmetric axis.

As shown in Fig M, the community transitions from specialist dominance to generalist dominance (Panel (a)), and then back to specialist dominance (Panel (b)), as μ_g increases.

Importantly, this transition is entirely explained by the relative magnitude of the growth-death ratio between the generalist and the specialists. As indicated by the top axes in Fig M, the generalist dominates the community if and only if its growth-to-death ratio exceeds that of the specialists. These results robustly support our core theoretical conclusion stated in the main text: regardless of the specific shape of the trade-off curve, the key determinant of competitive outcomes in fluctuating environments is the growth-death ratio.

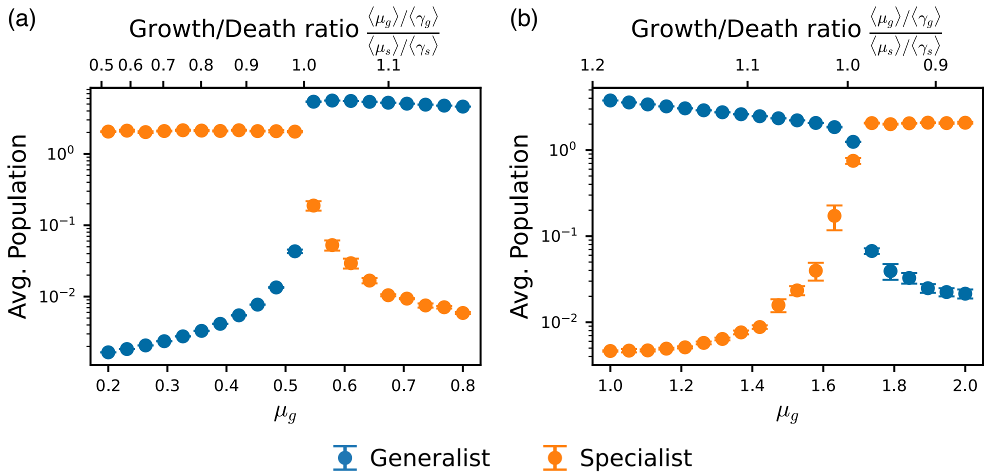

Fig M. Effect of increasing generalist growth rate on temporally averaged populations. The temporally averaged populations are shown as a function of the generalist's growth rate. The growth rates of the two specialists are fixed at $(\mu_A, \mu_B) = (0.25, 1.0)$ and $(1.0, 0.25)$, whereas the generalist has symmetric growth rates of (μ_g, μ_g). The two panels illustrate the results across different ranges of μ_g: (a) $0.2 \leq \mu_g \leq 0.8$, (b) $1.0 \leq \mu_g \leq 2.0$. All other parameters are identical to Fig 3 in the main text.

11 Simulations allowing access to sub-optimal interior phenotypes

In the main text model, phenotypes were strictly restricted to the trade-off curve ($\sqrt{\mu_A \mu_B} = \bar{\mu}$). In this section, we investigated how the evolutionary outcomes might change when phenotypes within the interior of the trade-off curve ($\sqrt{\mu_A \mu_B} \leq \bar{\mu}$) are also accessible. Specifically, we examine whether a species can gain a competitive advantage by lowering its growth rates below the trade-off boundary to decrease its death rate.

We first conducted numerical simulations where the strict boundary constraint is relaxed. We discretized the accessible region ($\sqrt{\mu_A \mu_B} \leq \bar{\mu}$) into a grid on a logarithmic scale, allowing phenotypic switching among adjacent grid points.

Our simulations reveal that under conditions with a sufficiently high $\bar{\mu}$, phenotypes located inside the sub-optimal region can indeed outcompete boundary phenotypes and become dominant (Fig N). However, in all such cases, the surviving interior phenotypes were exclusively generalists ($\mu_A = \mu_B$). Specialist-like strategies ($\mu_A \neq \mu_B$) were never observed to dominate within the interior region, suggesting that a specialist can only be optimal when it is located strictly on the trade-off boundary.

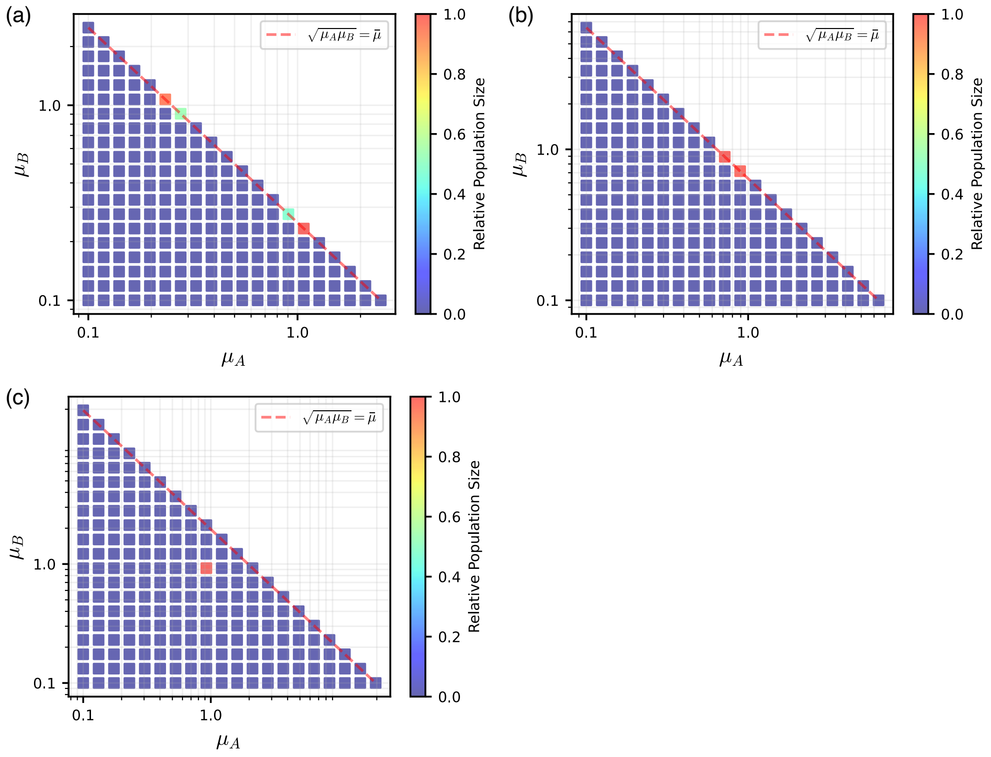

Fig N. Simulations allowing access to sub-optimal interior phenotypes. The panels show the simulation results for different values of $\bar{\mu}$: (a) $\bar{\mu} = 0.5$, (b) $\bar{\mu} = 0.8$, and (c) $\bar{\mu} = 1.4$. Each square corresponds to a single phenotype. The colormap indicates its relative population size, normalized such that the maximum time-averaged population size within the community is exactly 1. Simulations were run until 3.0×10^5, and population sizes were time-averaged over the last 20 nutrient supply intervals. At each nutrient supply event, one of the two nutrient types (A or B) is chosen at random. Phenotype switching occurs at an equal rate $p = 10^{-4}$ to each of the adjacent eight phenotypes. All other parameters are identical to those in Fig 5A in the main text.

To mathematically validate this observation, we evaluated the growth-to-death ratio $f(x,y)$ in the interior region. Let the specific growth rates in the two environments be $(\mu_A, \mu_B) = (x, y)$. The ratio is defined as:

$$f(x,y) = \frac{x+y}{a(\exp(bx) + \exp(by))}. \tag{75}$$

Consider an arbitrary interior specialist phenotype (x_0, y_0) where $x_0 \neq y_0$, located within the accessible region $x_0 y_0 \leq \bar{\mu}^2$. Let the sum of its growth rates be a constant C, such that $x_0 + y_0 = C$. By substituting $y = C - x$, we can express the growth-to-death ratio along the line $x + y = C$ as a single-variable function $f(x)$:

$$f(x) = \frac{C}{a(\exp(bx) + \exp(b(C-x)))}. \tag{76}$$

To maximize $f(x)$ for a given constant C, we must minimize its denominator, $g(x) = a(\exp(bx) + \exp(b(C-x)))$. The first and second derivatives of $g(x)$ are:

$$g'(x) = ab(\exp(bx) - \exp(b(C-x))), \tag{77}$$

$$g''(x) = ab^2(\exp(bx) + \exp(b(C-x))). \tag{78}$$

Setting $g'(x) = 0$ yields $x = C/2$. Because $g''(x) > 0$ for all real x, $g(x)$ is a strictly convex function with a unique global minimum at $x = C/2$. This strict convexity mathematically guarantees that $f(x)$ strictly and monotonically increases as x moves along the line $x + y = C$ towards the symmetric center $x = C/2$. Therefore, any phenotype on this line that is closer to the center $(C/2, C/2)$ will achieve a strictly higher growth-to-death ratio than the asymmetric specialist (x_0, y_0).

Finally, we evaluate whether these mathematically superior points closer to $(C/2, C/2)$ are accessible within our defined region ($xy \leq \bar{\mu}^2$):

1. If $C/2 \leq \bar{\mu}$: The perfectly symmetric generalist $(C/2, C/2)$ is accessible. Because it lies at the exact center, it achieves a strictly higher ratio than (x_0, y_0).

2. If $C/2 > \bar{\mu}$: The symmetric generalist $(C/2, C/2)$ lies outside the accessible region. Consequently, the line segment connecting (x_0, y_0) to the center must cross the trade-off boundary $xy = \bar{\mu}^2$ at an intermediate point (x_α, y_α). Because (x_α, y_α) lies strictly between (x_0, y_0) and the center, the monotonic increase of $f(x)$ guarantees that this boundary phenotype (x_α, y_α) has a strictly higher ratio than (x_0, y_0).

In either case, a strictly better phenotype—either an interior generalist or a boundary phenotype—always exists. Therefore, no interior specialist can ever maximize the growth-to-death ratio, perfectly aligning with our simulation results.

References

1. Caetano R, Ispolatov Y, Doebeli M. Evolution of Diversity in Metabolic Strategies. eLife. 2021 Aug;10:e67764. doi:10.7554/eLife.67764.

2. Monod J. THE GROWTH OF BACTERIAL CULTURES. Annual Review of Microbiology. 1949 Oct;3(Volume 3, 1949):371–394. doi:10.1146/annurev.mi.03.100149.002103.
